# Screening of Fenofibrate-Simvastatin Solid Dispersions in the Development of Fixed-Dose Formulations for the Treatment of Lipid Disorders

**DOI:** 10.3390/pharmaceutics15020603

**Published:** 2023-02-10

**Authors:** Agata Górniak, Hanna Czapor-Irzabek, Adrianna Złocińska, Agnieszka Gawin-Mikołajewicz, Bożena Karolewicz

**Affiliations:** 1 Laboratory of Elemental Analysis and Structural Research, Wroclaw Medical University, Borowska 211A, 50-556 Wroclaw, Poland; 2 Department of Drug Form Technology, Wroclaw Medical University, Borowska 211A, 50-556 Wroclaw, Poland

**Keywords:** simvastatin, fenofibrate, solid dispersion, eutectic, dissolution improvement, dyslipidemia, fixed-dose combination

## Abstract

The combination of statins and fibrates in the treatment of lipid abnormalities effectively regulates individual lipid fraction levels. In this study, the screening and assessment of the physicochemical properties of simvastatin-fenofibrate solid dispersions were performed. Fenofibrate and simvastatin were processed using the kneading method in different weight ratios, and the resulting solid dispersions were assessed using differential scanning calorimetry (DSC), X-ray powder diffractometry (XRPD), Fourier-transform infrared spectroscopy (FTIR), scanning electron microscopy (SEM), contact angle, as well as dissolution tests. The obtained results confirmed the formation of a simple eutectic phase diagram, with a eutectic point containing 79 wt% fenofibrate and 21 wt% simvastatin, lack of chemical interactions between the ingredients, and simvastatin impact on improving fenofibrate dissolution profile, due to the formation of crystalline solid dispersions by the kneading method.

## 1. Introduction

Fixed-dose combinations (FDCs) are defined as the combination of two or more active pharmaceutical ingredients (APIs) in a single dosage form [1]. With the population aging and lifestyle disease morbidity increasing, an increased interest in oral FDCs has been observed. They have been shown to offer a clinically important opportunity to simplify administration (due to a reduction in the number of medications) and improve patient adherence, which is particularly important in the treatment of chronic diseases such as cardiovascular disease (CVD), lipid disorders, and metabolic syndrome [2]. Moreover, the application of certain modifications improves the solubility and dissolution rate resulting in bioavailability improvements and a dose reduction effect [3]. As a result, FDC administration can improve the efficiency of pharmacotherapy and potentially reduces the number of resources needed for patients and the healthcare system [4].

Lipid disorders in the form of hypertriglyceridemia, high values of LDL-C, and low levels of HDL-C are common in elderly patients with type 2 diabetes and metabolic syndrome [5]. Pharmacological treatment of the above-mentioned lipid disorders begins with statin administration, and in cases they are ineffective, combination therapy with fibrates can be considered. FEN, as a prodrug, is metabolized in the liver to fibric acid, which, through its agonistic action on peroxisome proliferator-activated receptor-alpha (PPAR-α), can increase lipolysis, activate lipoprotein lipase, and synthesize the apolipoproteins A-I and A-II. Their mechanism of action translates directly into the lipid profile, in which, under the influence of FEN, a reduction in TG and LDL-C concentrations, as well as an increase of HDL-C, can be observed [6]. The primary target of hypercholesterolemia treatment is elevated low-density lipoprotein cholesterol (LDL-C) [7]. The goals for therapy also included high triglycerides (TG) and low concentrations of high-density lipoprotein cholesterol (HDL-C) [8]. Correcting these lipid disorders should be an integral part of antihypertensive therapy. Fibrates are an effective and well-tolerated treatment option for reducing TG and increasing HDL-C. They are recommended as an adjunct therapy for patients receiving statins when LDL-C or non-HDL-C does not reach target levels [9]. The combination of a statin and fibrate can raise the risk of myopathy and rhabdomyolysis [9]. However, the increase of myopathy risk (associated with the combination of particular statins and fibrates) is lowest with fenofibrate (FEN) [10]. Therefore, the combination of a statin and FEN appears to be the most appropriate choice for patients with atherogenic lipid profiles [9]. In recent years, various independent studies have been carried out (including DIACOR, SAFARI, and ACCORD) to evaluate the efficacy of FEN in combination with SIM in patients with mixed dyslipidemia, diabetes, and metabolic syndrome [11]. Clinical data indicate a benefit of combination therapy for SIM and FEN. Studies have shown that combination therapy leads to better improvements in total cholesterol (TC), TG, and HDL-C concentrations than SIM monotherapy and better concentrations of TC, LDL-C, and non-HDL-C than FEN monotherapy [12]. The introduction of combination therapy in the last decade has provided a greater opportunity in clinical practice to achieve LDL-C targets, especially in those that are at high cardiovascular risk, are refractory to statin monotherapy, and in patients who develop side effects with high doses of statins [13,14]. According to the current guidelines of the European Society of Cardiology (ESC), FEN does not inhibit the metabolism of statins and does not increase their concentrations; thus, does not indirectly intensify myopathy [15].

The first combined preparation containing a fibrate and statin appeared on the European market in 2011. The European Commission has issued a marketing authorization for Pravafenix^®^ that is valid throughout the European Union. This product contains 160 mg of FEN in combination with 40 mg of pravastatin sodium [16]. In 2013, the European Commission issued a marketing authorization for another, Cholib^®^ bi-component preparation. This product contains FEN in combination with SIM in two registered doses, 145 mg/20 mg or 145 mg/40 mg, respectively [17]. In the European Public Assessment Report, the results of TG levels in patients after 12 weeks of treatment with Cholib^®^ 145/20 mg compared to SIM 20 mg alone were reported. The TG levels decreased by approximately 36% in the Cholib^®^ group compared to 12% in the SIM group. In addition, HDL-C levels increased by approximately 7% with the use of Cholib^®^ and approximately 2% with the use of SIM. Cholib^®^ 145/40 mg was also compared to SIM 40 mg, and a greater reduction in TG was seen with the combined dosage form (33% in the Cholib^®^ group compared to 7% in the SIM group). Additionally, an increase of HDL-C (6% in the Cholib^®^ group compared to 1% in the SIM group) was observed. Cholib^®^, in comparison with other statins, i.e., atorvastatin and pravastatin, has been shown to be more effective than the respective statins when given alone [18].

Despite the availability of fixed-dose products containing SIM and FEN, there is a lack of information in the available literature about the formation of solid dispersions (SDs). Recent studies have shown that FEN and SIM form a simple eutectic system [19]. Eutectic mixtures, classified as 1-st generation SDs, are often characterized by improved dissolution profiles of their components [20]. This property may prove beneficial in oral FDCs, which are becoming increasingly popular in the treatment of diseases that require taking several medications at the same time every day, such as CVD, type 2 diabetes, and metabolic syndrome.

In this study, the kneading method has been applied to obtain SDs containing two poorly water-soluble drugs, FEN and SIM. Both of these APIs are classified into the II class of the Biopharmaceutical Classification System (BCS) [21,22]. FEN is practically insoluble in aqueous media (0.1 μg/mL) [23], while the solubility of SIM in water is approximately three orders of magnitude higher (30 μg/mL) [24]. The purpose of this work was to examine physicochemical properties as well as to assess the possibility of dissolution improvement due to the formation of crystalline FEN-SIM SDs. These screening studies may contribute to the further development of an oral fixed-dose formulation containing both APIs with an improved FEN dissolution profile.

## 2. Materials and Methods

### 2.1. Materials

**Figure 1:**
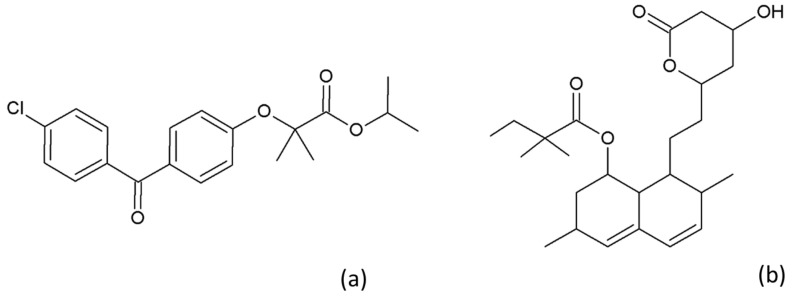
Chemical structures of (**a**) fenofibrate and (**b**) simvastatin.

FEN with a 99% purity (Figure 1a) was purchased from Sigma-Aldrich (St. Louis, MO, USA). SIM (Figure 1b) was kindly donated by Polpharma (Gdańsk, Poland). Sodium lauryl sulfate (SLS) was supplied from Sigma-Aldrich (Schnelldorf, Germany) and used in the preparation of the dissolution medium. Ethanol (99.8%, pure p.a.) was purchased from Avantor Performance Materials (Gliwice, Poland) and was used as a solvent in the preparation of the SDs. Phosphoric acid (85%) was purchased from Chempur (Piekary Śląskie, Poland), and acetonitrile (high-performance liquid chromatography (HPLC) grade) was purchased from Merck (Darmstadt, Germany).

### 2.2. Fenofibrate-Simvastatin Solid Dispersions Preparation

FEN-SIM SDs in weight ratios of 10:90, 20:90, 30:70, 40:60, 50:50, 60:40, 70:30, 75:25, 80:20, 90:10, 93:7, 95:5, and 97:3 *w*/*w*, respectively, were prepared using the kneading method. For this purpose, the accurately weighed quantities of each component (1 g of summarized mixture weight) were wetted with 1 mL of ethanol and mixed by grinding for about 15 min until the solvent completely evaporated. The prepared mixtures were transferred into tightly closed amber glass containers and stored in a desiccator until the tests were conducted. The compositions of the obtained dispersions are summarized in Table 1.

### 2.3. Drug Content

A quantity of 5 mg of each prepared mixture was accurately dissolved in 100 mL of HPLC-grade methanol. The content of FEN and SIM in the obtained solutions was determined using the HPLC method described in Section 2.10 and presented in Table 1.

### 2.4. Scanning Electron Microscopy

The morphology of the prepared mixtures as well as the pure ingredients, was evaluated using a Sigma 500 VP scanning electron microscope (Zeiss, Jena, Germany). To improve the discharge process, just before measurements, the investigated samples were covered with a layer of gold using a Quorum machine (Quorum International, Fort Worth, TX, USA).

### 2.5. Thermal Analysis

Differential scanning calorimetry (DSC) measurements were carried out on a DSC 214 Polyma instrument (Netzsch, Selb, Germany), which was calibrated using indium (156.6 °C), tin (231.9 °C), bismuth (271.4 °C), and zinc (419.5 °C) as the standards. Samples of pure constituents and each SDs (4–5 mg) were weighed in standard Al crucibles (40 µL) covered by a lid. The lid had one hole to ensure the flow of dry nitrogen (purity 99.999%) purged through the measurement chamber at a constant rate of 25 mL/min. An empty crucible with a punched lid was used as a reference. The heating curves were recorded between the temperature range of 25–150 °C with a constant rate of 5 °C/min. To observe the thermal effect associated with the eutectic reaction, DSC experiments for some of the samples were performed between the temperature range of 60–90 °C using a heating rate of 0.5 °C/min. Measurements were performed in triplicate, and the mean values were calculated. 

### 2.6. X-ray Powder Diffraction

XRPD studies were performed to identify the solid phases and to verify the crystalline nature of the investigated samples. X-ray diffraction patterns were collected at ambient temperature using a D2 Phaser (Bruker AXS, Karlsruhe, Germany) powder diffraction system equipped with a horizontal goniometer operating in the 2θ mode. An LYNXEYE detector and CuKα radiation tube (operating at 30 kV and 10 mA) were used. The instruments were calibrated with a corundum standard supplied by Bruker AXS. Samples were scanned over a 2θ range of 5° to 40° with a step size of 0.02° and a 1-s exposure time per step using a low-background holder. The XRPD patterns were analyzed using Diffrac.Eva V3.2 (Bruker AXS, Karlsruhe, Germany) software.

### 2.7. Fourier-Transform Infrared Spectroscopy

The FTIR spectra were collected using a Nicolet iS50 FTIR (Thermo Scientific, Waltham, MA, USA) spectrometer on an attenuated total reflection (ATR) module equipped with a diamond crystal. Data collection and analysis were achieved using OMNIC software version 5.0. The spectra were recorded in wavenumber ranges from 4000 cm^−1^ to 400 cm^−1^ with a resolution of 4 cm^−1^ and an obtained accumulation mean of 32 scans per sample.

### 2.8. Contact Angle

The contact angle was measured using a Contact Angle Goniometer (Osilla Ltd., Sheffield, UK). For this purpose, a 5 mm diameter drop of pure water was deposited on the sample surface with a precision syringe with a 110 μm inner diameter (Agilent Technologies Inc., Santa Clara, CA, USA). The deposition of the drop was recorded in the form of a short movie (20 frames per second) using a high-resolution camera (1920 × 1080 pixels). The contact angle was estimated using the selected film frame immediately after drop deposition on the powder surface. 

### 2.9. Dissolution Tests

#### 2.9.1. Intrinsic Dissolution Rate Method

Intrinsic dissolution rate (IDR) studies were carried out for the FEN-SIM SDs, as well as for individual components (FEN and SIM). An appropriate system was fitted with an SR8-PLUS dissolution bath (Hanson Research, Chatsworth, CA, USA) and a 7-channel peristaltic pump. The dissolution rate was determined using the pharmacopoeial rotating disk method. The dissolution medium was composed of 900 mL of a 0.5% SLS solution prepared using deionized and degassed pure water. Accurately weighed 100 mg samples of the prepared SDs and pure APIs were pressed using a die with a hole diameter of 0.8 cm for 1 min under a pressure of 1 ton using a laboratory hydraulic press. The dissolution test was performed at 37 ± 0.5 °C with a rotating disc speed of 50 rpm. Aliquots of 3 mL were withdrawn using a 45 μm cannula filter at 2, 4, 6, 8, 10, 12, 15, 18, 21, and 24 h. The concentrations of both components in the collected samples were determined using the HPLC method immediately after sampling.

#### 2.9.2. Paddle Method

The paddle dissolution tests were performed for selected FEN-SIM SDs as well as for the individual components (FEN and SIM) using the USP 2 pharmacopoeial method. Studies were carried out using a Varian VK 7025 apparatus (Varian, Palo Alto, CA, USA). Accurately weighed 100 mg samples of the prepared SDs and pure APIs were introduced into vessels containing 900 mL of 0.5% SLS solution. The dissolution test was performed at 37 ± 0.5 °C and a paddles speed of 50 rpm. To determine the amount of APIs released, 3 mL aliquots were taken at a sampling time of 5, 10, 20, 30, 40, 50, 60, 90, and 120 min. During collection, the samples were filtered with a pore size of 45 µm cannula filters. The concentrations of both APIs were determined by means of the HPLC method immediately after sampling.

### 2.10. High-Performance Liquid Chromatography

Concentrations of FEN and SIM were determined using a separation module HPLC Dionex Ultimate 3000 (Thermo Scientific, Waltham, MA, USA) with a UV-DAD detector. Diluted samples were injected into a C18 column (Purosphere^®^, RP-18 125 mm × 3 mm, 5 μm, Merck, Germany). FEN and SIM were eluted with a mobile phase of acetonitrile:water at ratios (by volume) of 70:30, at a constant flow rate of l.2 mL/min, and then quantified with UV detection. The detection wavelengths for FEN and SIM were 286 and 238 nm, respectively. The retention times were 2.3 min for SIM and 3.4 min for FEN. A standard curve was determined for each drug tested. Linearity of the curves was observed at a concentration range of approximately 0.208–207.8 µg/mL for FEN and 0.199–199 µg/mL for SIM with a correlation coefficient of r^2^ > 0.999 determined by linear regression analysis.

## 3. Results and Discussion

### 3.1. Phase Transitions Studies

**Figure 2:**
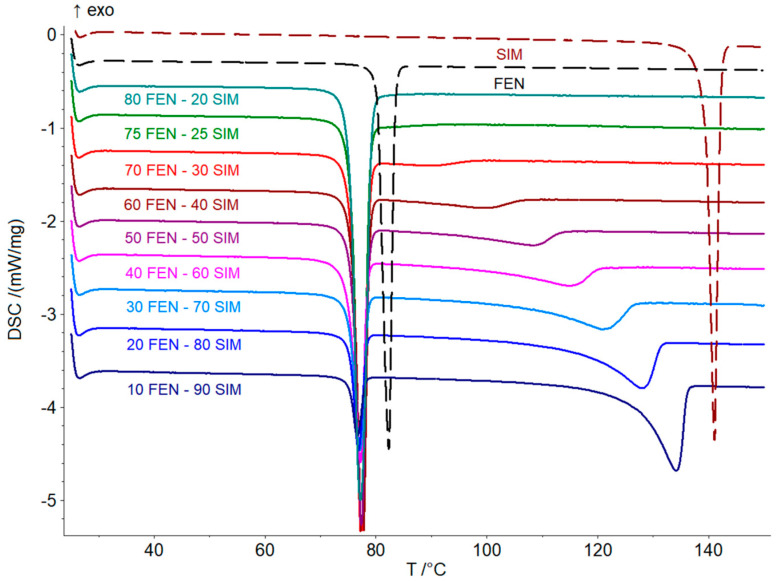
DSC curves of FEN, SIM, and FEN-SIM SDs containing 10–80 FEN wt% obtained at the heating rate of 5 °C/min in a temperature range from 25 to 150 °C.

**Figure 3:**
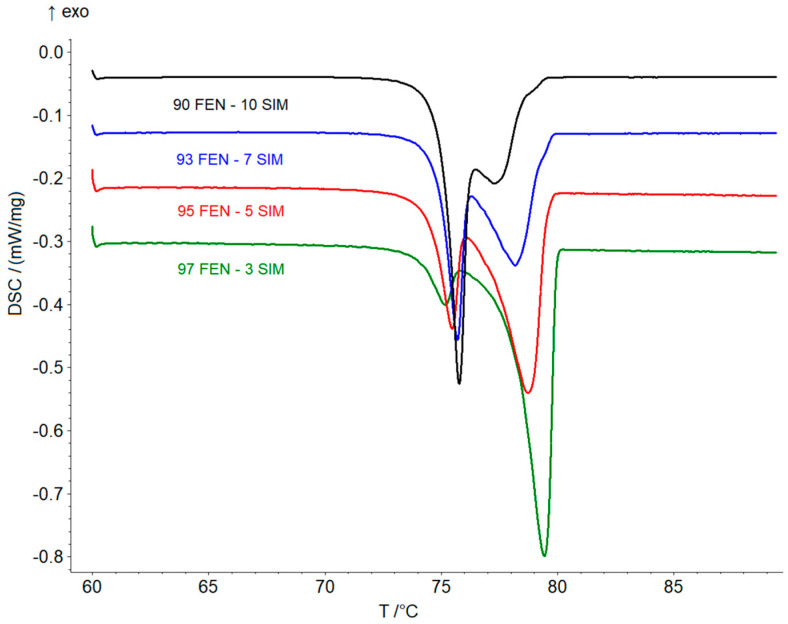
DSC curves of FEN-SIM SDs containing 90–97 FEN wt% obtained at the heating rate of 0.5 °C/min in a temperature range from 60 to 90 °C.

**Figure 4:**
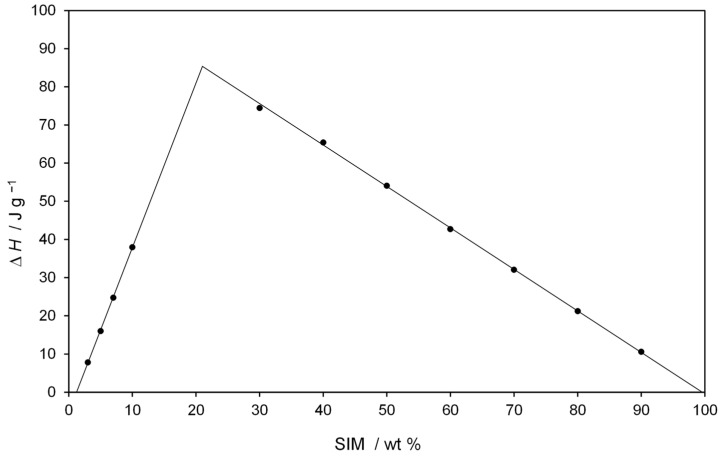
Tamman’s triangle of eutectic reaction enthalpy ΔH at 74.8 °C versus SIM wt%.

**Figure 5:**
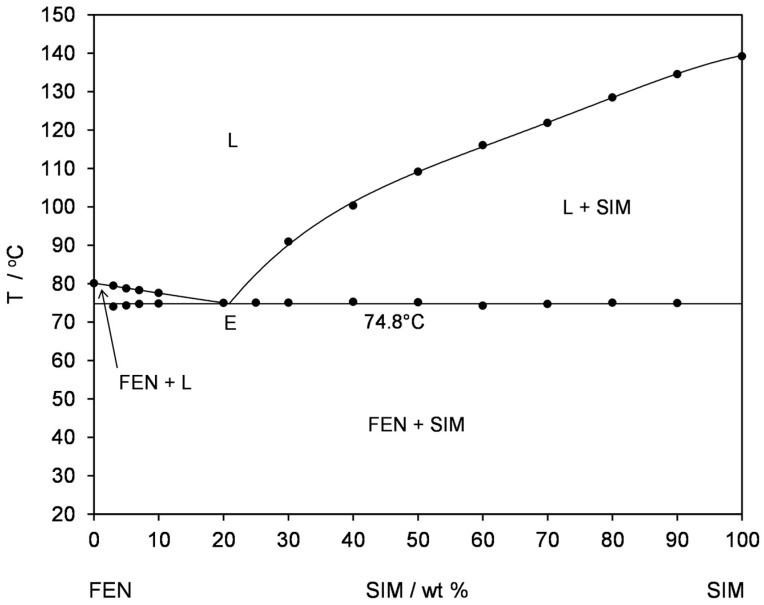
Temperature–composition phase diagram for the FEN-SIM binary system: (L) liquid, (L + SIM) liquid + solid SIM, (FEN + L) solid FEN + liquid, (E) eutectic point, (FEN + SIM) solid FEN + solid SIM.

Thermal studies were conducted to confirm the formation of a eutectic mixture in the FEN-SIM system, first reported by Knapik-Kowalczuk et al. [19]. There were two endothermic effects observed on DSC heating curves (Figure 2) for most samples of FEN-SIM SDs. The first one appeared at a constant onset temperature near 74.8 °C and had a variable area that depended on the mixture composition. This peak was related to the eutectic reaction: solid FEN + solid SIM = liquid (L), and for samples containing more than 80 wt% of FEN were observed on DSC curves registered at a heating rate of 0.5 °C/min (Figure 3). The value of eutectic reaction enthalpy changes with sample composition (Table 2) and formed a characteristic Tamman’s triangle (Figure 4) with a maximum solid dispersion containing 79 wt% FEN and 21 wt% SIM as a eutectic point, which corresponded well with the phase diagram (Figure 5) as well as with previous observations [19]. The value of eutectic reaction enthalpy approaches zero, close to pure FEN and SIM, which indicates the absence of solid solutions in the FEN-SIM system.

### 3.2. Phase Composition Analysis by X-ray Powder Diffraction

**Figure 6:**
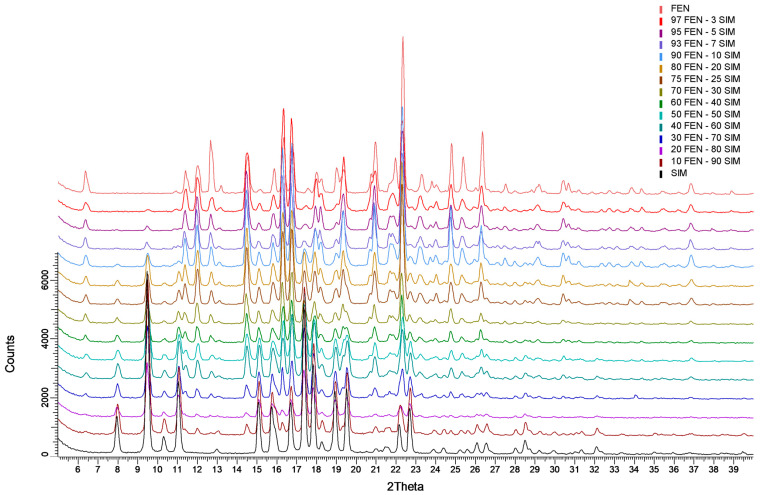
XRPD patterns of FEN, SIM, and FEN-SIM SDs.

The analysis of XRPD patterns (Figure 6) revealed a crystalline nature of the raw components (FEN and SIM), as well as the examined FEN-SIM SDs. The FEN pattern had characteristic reflections at angular positions 2θ: 11.4°, 12.0°, 12.7°, 14.6°, 16.4°, 22.4°, 24.8°, and 26.4° characteristic of its first polymorphic form [25]. Whereas, the SIM pattern showed distinctive reflections at 2θ values of 8.0°, 9.5°, 11.0°, 15.1°, 17.4°, and 22.7°, characteristic for the orthorhombic crystalline form stable at room temperature [26]. The XRPD patterns of the SDs have revealed only reflections characteristic of the individual components. No additional reflections were detected, which excludes the presence of phases other than the crystalline phases of the primary constituents.

### 3.3. Infrared Spectroscopy Analysis

**Figure 7:**
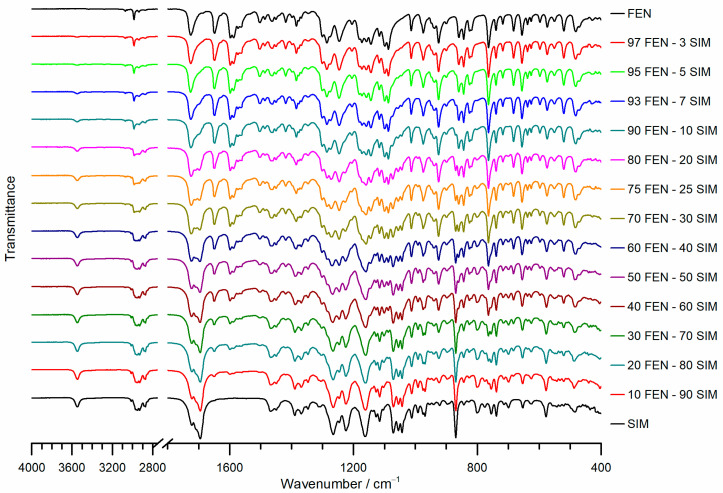
FTIR spectra of pure FEN, SIM, and FEN-SIM binary SDs.

The FTIR spectra of raw FEN, SIM, and FEN-SIM SDs were reported in Figure 7. The FTIR spectrum of FEN showed main absorption bands, significant to C–H stretching of isopropyl group at 2984 cm^−1^, ester carbonyl stretching at 1725 cm^−1^, carbonyl stretching at 1648 cm^−1^, benzene ring stretching at 1597 cm^−1^, and aryl ether at 1286 cm^−1^ [27]. Whereas the FTIR spectrum of SIM showed main absorption bands, significant to free O–H stretching vibration at 3547 cm^−1^, aliphatic C–H vibrations at 2951 cm^−1^, 2930 cm^−1^, 2872 cm^−1^, and 1467 cm^−1^, ester C=O vibration at 1695 cm^−1^ and 1266 cm^−1^, and ester and lactone C-O-C vibrations at 1162 cm^−1^ [28]. FTIR spectra of FEN-SIM SDs appeared as the superimposition of bands significant for both components, and their intensities were related to the drug-drug ratio. Neither new bands nor shifts of the bands representative of SIM or FEN were observed. Consequently, any kind of chemical interaction between the constituents of the prepared SDs should be excluded.

### 3.4. Shape Morphology

**Figure 8:**
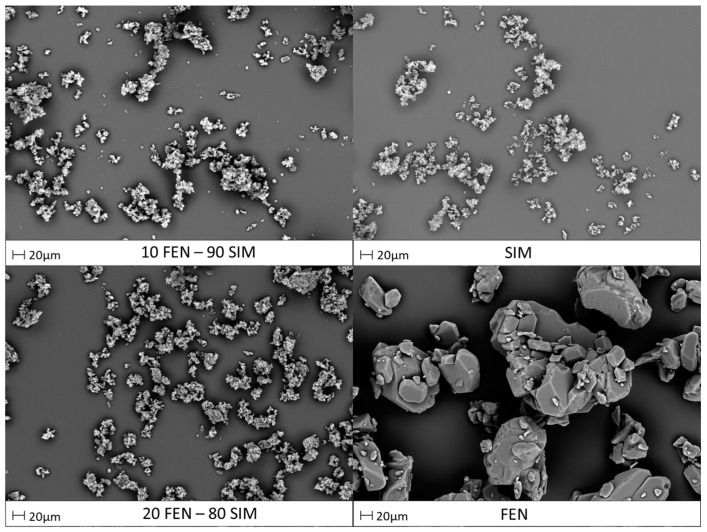
SEM images of raw FEN and SIM and representative FEN-SIM samples.

SEM images presented in Figure 8 show the morphology of primary constituents and representative FEN-SIM SDs. Pure FEN revealed a polyhedral shape crystalline structure, whereas the morphology of pure SIM was characterized by finer crystals. The particle size estimation, according to the specified scale bar, has revealed that pure SIM particles were smaller (<10 μm) compared with that of pure FEN (>20 μm). The SEM images of SDs revealed fine particles with a crystalline nature, which was also observed in XRPD studies. The kneading method affected the size and morphology of prepared dispersions, causing an increase of homogeneity and significant particle size reduction.

### 3.5. Dissolution Studies

FEN, as an API belonging to BCS class II, is used, depending on the clinical indications, as monotherapy in doses ranging from 30 mg to 200 mg. Solid dosage forms are available as tablets in doses of 40–160 mg, capsules in doses of 50–267 mg, and capsules containing micronized API in doses of 30–267 mg. The Summary of Product Characteristics states that 100 mg of standard FEN once daily corresponds to 67 mg of micronized FEN. FEN is also available in combination with statins, including SIM, which are used to treat complex lipid disorders. The fixed FEN and SIM combination forms contain 140 mg of FEN and SIM in doses of 20–40 mg. Therefore, in this work, screening studies were undertaken to cover the full range of weight ratios of both APIs to assess the mutual effect on their physicochemical properties, including dissolution rates. The USP monographs recommend 0.05 M and 0.025 M aqueous SLS solution as a dissolution medium for fenofibrate capsules and tablets, respectively, and for simvastatin tablets, a phosphate buffer solution containing 0.5% SLS [29]. The 0.5% SLS solution was also selected by Anumolu et al. [30] as a discriminative dissolution medium for fenofibrate-atorvastatin formulations. Their screening study included dissolution tests in 18 different media, i.e., simulated gastric fluid without enzyme (SGF), simulated intestinal fluid (SIF), blank fasted state simulated intestinal fluid (BFaSSIF), blank fed state simulated intestinal fluid (BFeSSIF,), modified fasted state simulated intestinal fluid, modified fed state simulated intestinal fluid. Anumolu et al. summarized that fenofibrate release was less than 2% in SGF, SIF, BFaSSIF, and BFeSSIF, in comparison to about 50% for 0.5% SLS medium [30]. Considering the above and significant differences in solubility between FEN and SIM, we recognized 0.5% SLS aqueous solution as the optimal discriminative medium to ensure the solubility of both APIs.

**Figure 9:**
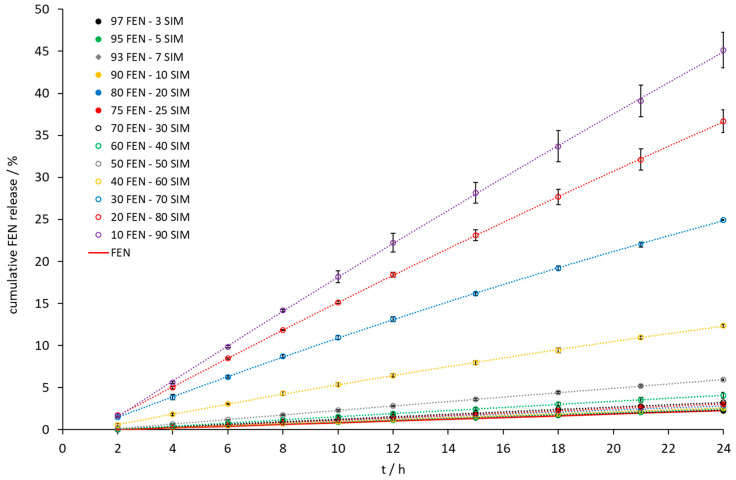
The dissolution profile of FEN released from FEN-SIM samples on IDR studies.

**Figure 10:**
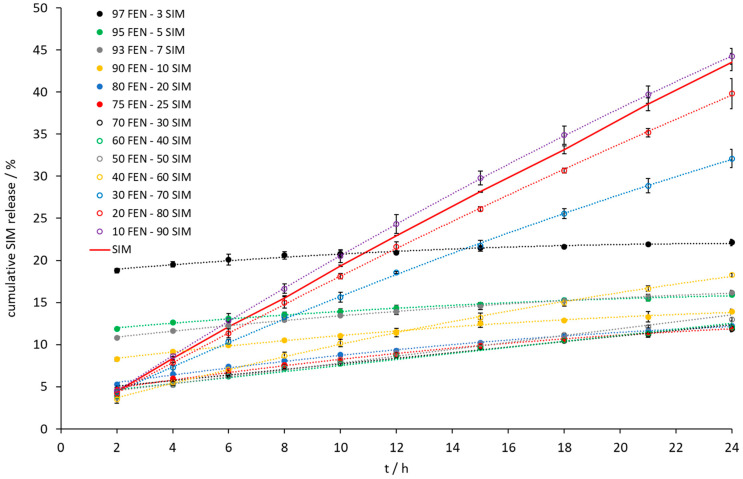
The dissolution profile of SIM released from FEN-SIM samples on IDR studies.

Intrinsic dissolution tests showed an increase of the dissolution rate of FEN released from all tested SDs (Figure 9). A significant increase was observed when the samples contained a 10 to 40 wt% of FEN. The IDR test (Figure 9 and Figure 10) revealed the fastest dissolution of both active ingredients occurred at a composition of 10 wt% FEN and 90 wt% SIM. Thermal studies confirmed eutectic formation with a composition of 79.0 FEN-21.0 SIM, which may affect the dissolution of both APIs. After 24 h of the IDR test, only a two-fold increase of the amount of dissolved FEN was observed for the 80.0 FEN-20.0 SIM sample, with a composition close to the eutectic point. The IDR test enables the comparison of dissolution rates of APIs. However, during the examination, only one surface of the pressed formulation has contact with the dissolution medium, which does not take into account the expanded surface of obtained formulations. In SDs, such as eutectic mixtures, the expanded surface of particles plays a crucial role during contact with the dissolution medium. Therefore, to observe the influence of eutectic formation on the dissolution profiles of both APIs, the paddle method was applied in this study. The paddle method tests are advantageous in the analysis of powdered drugs enclosed in capsule form. For paddle tests, we selected the 10.0 FEN-90.0 SIM SD, which best dissolves FEN according to the IDR test, as well as the 80.0 FEN-20.0 SIM sample closest to the eutectic point and we prepared the SD 79.0 FEN-21.0 SIM corresponding to the eutectic point composition.

**Figure 11:**
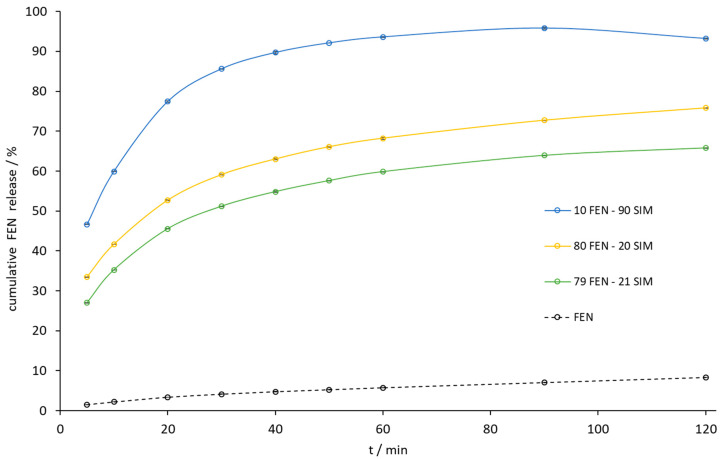
The dissolution profile of FEN released from FEN-SIM samples by the paddle method.

**Figure 12:**
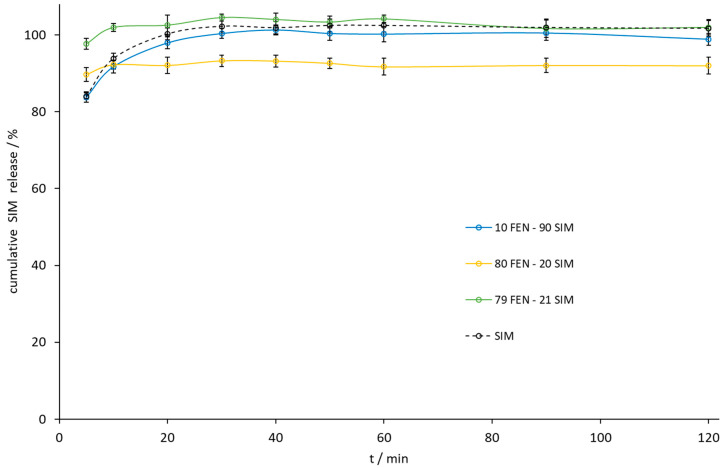
The dissolution profile of SIM released from FEN-SIM samples by the paddle method.

The paddle method dissolution profile of FEN released from the 10.0 FEN-90.0 SIM SD was the best, but the amount of FEN released during the dissolution of the eutectic mixture increased 10-fold compared to a similar release profile (Figure 11). Especially noticeable was the dissolution rate improvement at the initial time of the test. Within the first 5 min, the eutectic mixture released 27% of FEN compared to 2% for the pure API sample. During this time, the eutectic mixture also released almost 100% of the SIM (Figure 12).

The results observed with the paddle method revealed that the formation of FEN-SIM eutectic SD had a real impact on improving FEN dissolution, and the kneading could be an effective method in the preparation of the SDs.

### 3.6. Contact Angle

**Figure 13:**
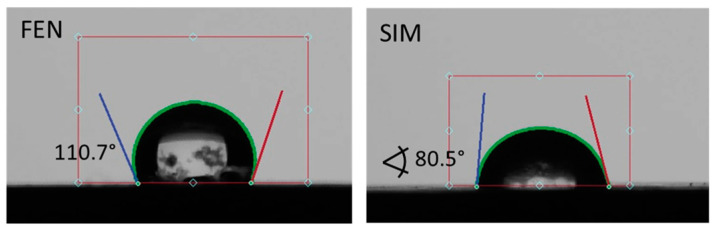
The contact angle of water on the FEN and SIM powder surfaces.

**Figure 14:**
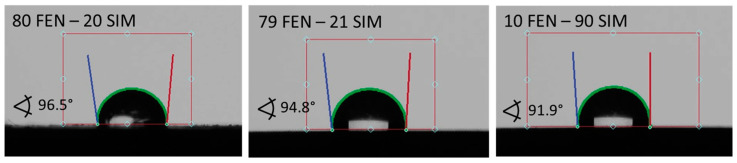
The contact angle of water on the powder surfaces of SDs tested with the paddle method.

Contact angles observed for pure APIs and SDs subjected to paddle dissolution tests are shown in Figure 13 and Figure 14, respectively. The comparison of obtained results showed that the examined SDs are characterized by a better wettability than pure FEN and slightly worse than pure SIM. Such observations correspond well with dissolution studies and confirm the positive influence of SD formation on FEN dissolution properties.

## 4. Conclusions

In recent years, fixed-dose preparations containing two or more APIs in a single dosage form have been increasing in popularity for the treatment of lipid disorders. Due to the prevalence of lipid disorders, the use of FDCs using fibrates and statins is the subject of many preformulation studies and the development of this type of medicinal product. Preparation of drug-drug SD may be a simple and good solution leading to improved dissolution profiles of poorly water-soluble ingredients in FDCs. Excluding drug-drug chemical interactions between active ingredients play a crucial role in the preparation of such dispersions. In this study, the occurrence of chemical interactions between SIM and FEN in the solid state has been studied and excluded by means of infrared spectroscopy and XRPD. DSC studies confirmed that FEN forms a simple binary eutectic system with SIM. According to our research, the FEN-SIM eutectic mixture contains 79.0 wt% of FEN and 21.0 wt% of SIM. Taking into account the quantitative composition of the bi-component preparations available in tablet form, containing 145 mg of FEN and 20–40 mg of SIM, the presented results indicate the possibility of developing FDC formulation for oral delivery based on FEN-SIM eutectic SD. The analysis of dissolution results, especially with regard to FEN, shows that the eutectic SD can be incorporated into hard capsule formulations with an improved FEN dissolution profile. However, further in vivo studies are required to confirm the impact of dissolution enhancement on the oral bioavailability of studied APIs and their effectiveness in improving lipid profiles.

## Figures and Tables

**Table 1:** Compositions of FEN-SIM dispersions and APIs average content.

Sample Name	Composition/wt%		API Content/%	
	FEN	SIM	FEN	SIM
97 FEN–3 SIM	97.0	3.0	100.55 ± 0.20	123.20 ± 0.44
95 FEN–5 SIM	95.0	5.0	100.58 ± 0.12	121.19 ± 0.18
93 FEN–7 SIM	93.0	7.0	95.38 ± 0.06	105.91 ± 0.10
90 FEN–10 SIM	90.0	10.0	99.06 ± 0.04	105.00 ± 0.19
80 FEN–20 SIM	80.0	20.0	99.33 ± 0.08	103.61 ± 0.17
75 FEN–25 SIM	75.0	25.0	99.09 ± 0.12	100.74 ± 0.09
70 FEN–30 SIM	70.0	30.0	99.26 ± 0.17	99.40 ± 0.11
60 FEN–40 SIM	60.0	40.0	100.21 ± 0.12	99.83 ± 0.05
50 FEN–50 SIM	50.0	50.0	99.84 ± 0.05	99.51 ± 0.10
40 FEN–60 SIM	40.0	60.0	102.89 ± 0.24	99.43 ± 0.14
30 FEN–70 SIM	30.0	70.0	101.29 ± 0.06	100.38 ± 0.03
20 FEN–80 SIM	20.0	80.0	99.33 ± 0.08	99.54 ± 0.08
10 FEN–90 SIM	10.0	90.0	99.98 ± 0.34	100.49 ± 0.24

Data expressed as mean ± SD (*n* = 3).

**Table 2:** Experimental temperature and enthalpy values for phase transitions observed in the FEN-SIM system.

Sample Name	Composition/wt%		Eutectic Invariant		Liquidus Temperature/°C
	FEN	SIM	Temperature/°C	ΔH/J g^−1^	
FEN	100.0	0.0	-	-	80.1 ± 0.0
97 FEN–3 SIM	97.0	3.0	74.1 ± 0.2	7.8 ± 1.0	79.5 ± 0.1
95 FEN–5 SIM	95.0	5.0	74.3 ± 0.1	16.0 ± 0.3	78.8 ± 0.0
93 FEN–7 SIM	93.0	7.0	74.7 ± 0.1	24.7 ± 2.0	78.3 ± 0.1
90 FEN–10 SIM	90.0	10.0	74.9 ± 0.1	38.0 ± 1.1	77.6 ± 0.0
80 FEN–20 SIM	80.0	20.0	75.0 ± 0.1	-	-
75 FEN–25 SIM	75.0	25.0	75.1 ± 0.1	-	-
70 FEN–30 SIM	70.0	30.0	75.1 ± 0.4	74.5 ± 3.6	91.0 ± 0.5
60 FEN–40 SIM	60.0	40.0	75.3 ± 0.3	65.4 ± 0.3	100.4 ± 0.2
50 FEN–50 SIM	50.0	50.0	75.2 ± 0.4	54.1 ± 0.2	109.2 ± 0.2
40 FEN–60 SIM	40.0	60.0	74.3 ± 0.1	42.7 ± 0.7	116.1 ± 0.2
30 FEN–70 SIM	30.0	70.0	74.7 ± 0.2	32.1 ± 0.9	121.9 ± 0.1
20 FEN–80 SIM	20.0	80.0	75.1 ± 0.2	21.2 ± 0.1	128.5 ± 0.2
10 FEN–90 SIM	10.0	90.0	75.0 ± 0.2	10.6 ± 0.1	134.6 ± 0.1
SIM	0.0	100.0			139.2 ± 0.3

## Data Availability

Not applicable.
